# Neuroanatomy of reduced distortion of body-centred spatial coding during body tilt in stroke patients

**DOI:** 10.1038/s41598-023-38751-0

**Published:** 2023-07-22

**Authors:** Keisuke Tani, Shintaro Iio, Masato Kamiya, Kohei Yoshizawa, Takashi Shigematsu, Ichiro Fujishima, Satoshi Tanaka

**Affiliations:** 1 Laboratory of Psychology, Hamamatsu University School of Medicine, Hamamatsu, Shizuoka 431-3192 Japan; 2 Faculty of Psychology, Otemon Gakuin University, 2-1-15 Nishi-Ai, Ibaraki, Osaka 567-8502 Japan; 3 Department of Rehabilitation, Hamamatsu City Rehabilitation Hospital, Hamamatsu, Shizuoka 433-8511 Japan; 4 Department of Rehabilitation Medicine, Hamamatsu City Rehabilitation Hospital, Hamamatsu, Shizuoka 433-8511 Japan

**Keywords:** Human behaviour, Perception, Neurological disorders

## Abstract

Awareness of the direction of the body’s (longitudinal) axis is fundamental for action and perception. The perceived body axis orientation is strongly biased during body tilt; however, the neural substrates underlying this phenomenon remain largely unknown. Here, we tackled this issue using a neuropsychological approach in patients with hemispheric stroke. Thirty-seven stroke patients and 20 age-matched healthy controls adjusted a visual line with the perceived body longitudinal axis when the body was upright or laterally tilted by 10 degrees. The bias of the perceived body axis caused by body tilt, termed tilt-dependent error (TDE), was compared between the groups. The TDE was significantly smaller (i.e., less affected performance by body tilt) in the stroke group (15.9 ± 15.9°) than in the control group (25.7 ± 17.1°). Lesion subtraction analysis and Bayesian lesion-symptom inference revealed that the abnormally reduced TDEs were associated with lesions in the right occipitotemporal cortex, such as the superior and middle temporal gyri. Our findings contribute to a better understanding of the neuroanatomy of body-centred spatial coding during whole-body tilt.

## Introduction

The body (longitudinal) axis plays a fundamental role in space and in the perception of external events as an egocentric (head- or body-centred) reference frame^1–4^. Thus, accurate awareness of the direction of the body axis, regardless of the posture or body orientation in space, is crucial. Internal estimates of the body axis orientation involve the integration of multisensory information from the visual, vestibular, and somatosensory systems (see Ref.^5^ for a review). This afferent sensory information seems to be combined and integrated in the brain, likely in a weighted fashion, to reduce sensory ambiguity^6–8^. Nevertheless, the estimation of body axis orientation is strongly influenced by the body tilt orientation relative to gravity. More specifically, the subjective visual body axis (SVBA) is assessed by the participants’ adjustment of a visual line along the perceived body axis biases in the direction of the whole-body roll tilt^4,9–17^. The exact mechanism underlying this phenomenon remains unclear, but one possibility has been proposed: disturbance of head/body-centred spatial coding caused by changes in sensory input related to body tilt^11,18^.

Which brain region is responsible for the perceptual distortion of body axis orientation induced by whole-body tilt? In a recent study^16^ on healthy volunteers, we addressed this question by exploring the brain regions where the local grey matter volume was correlated with interindividual performance regarding the tilt-dependent errors (TDE) in the SVBA task using a voxel-based morphometry (VBM) analysis. A significant correlation was observed between TDE and grey matter volume in the bilateral occipitotemporal cortex, with peak voxels in the right middle occipital gyrus (MOG), which provides neuroanatomical insight into the mechanism of body-centred spatial coding during whole-body tilt. However, since no other studies have examined this issue, likely due to methodological limitations (e.g., difficulty in manipulating body orientation in space for classical neuroimaging methods), experimental evidence remains far from fully established. In addition, it should be noted that VBM studies can provide only correlational relationships between brain structure and behaviour^19^, and thus the causal relationship between brain regions and tilt-dependent SVBA bias remains unclear. Here, we attempted to address the above-mentioned challenges by employing a neuropsychological approach in patients with brain injury. This approach requires that brain injury precede behavioural measurements (the SVBA task in this case), thus allowing us to infer a more direct association between the identified brain regions and the perceptual distortion of body axis orientation during body tilt. In the present study, we quantified the tilt-dependent SVBA bias in patients with hemispheric stroke and age-matched healthy controls and explored the lesion location and extension specific to patients with abnormal SVBA bias using lesion analysis.

It also remains unclear whether perceptual distortion of body axis orientation during whole-body tilt is related to postural ability. Some studies have shown a significant correlation between the perceived body axis orientation in the supine position and weight-bearing asymmetry in standing for hemispheric stroke patients^1,20^, indicating a possible role of the perceived body axis in *static* postural ability. However, no studies have investigated whether changes in the perception of body axis orientation induced by body tilt relate to *dynamic* postural control. Dynamic and stable control of posture against gravity may be built on an accurate perception of the direction of the body axis (egocentric reference frame) and the gravitational axis (gravity-based reference frame), especially in situations where those axes are spatially dissociated. Hence, the secondary purpose of this study was to investigate whether the individual amplitude of tilt-dependent SVBA bias is related to dynamic postural ability in patients with stroke.

## Results

### Tilt-dependent SVBA bias in the stroke and control groups

**Figure 1:**
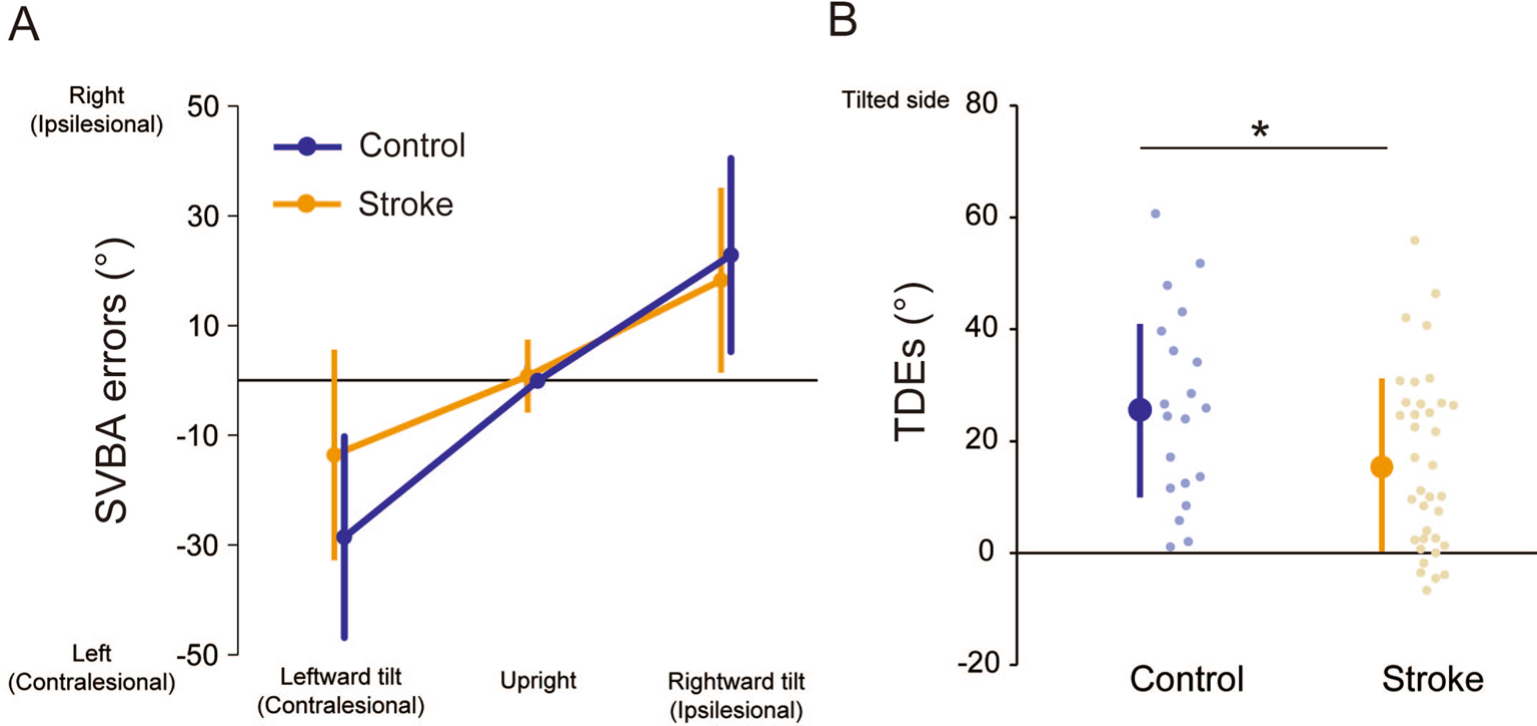
(**A**) Group-mean SVBA errors at each tilt position.in each group. (**B**) Group-mean TDEs in each group. Each light- and dark-coloured dots represent individual and group-mean data, respectively. Error bars represent the standard deviation (SD). *p < 0.05. Data to reproduce these figures are included in Supplementary Data.

We compared the perceptual distortion of body axis orientation between the stroke patients and healthy controls. Figure 1A shows the estimation errors of the SVBA task for each body angle in the control and stroke groups. We first checked whether the SVBA deviated significantly from the actual body axis orientation in the upright position in each group. Mean (± standard deviation: SD) SVBA errors were -0.3 ± 2.6° and 0.8 ± 6.6° in the control and stroke groups, respectively. One-sample t-tests (two-tailed) revealed that the SVBA errors in both groups did not differ significantly from zero (control, *t*_19_ = -0.19, *p* = 0.85; patient, *t*_36_ = 0.74, *p* = 0.47), indicating that the patients with stroke could accurately estimate the body axis orientation at upright as well as healthy controls.

We assessed the differences in SVBA bias induced by body tilt between the groups. In contrast to the upright position, the SVBA errors increased when the body was tilted in both groups, which is consistent with the results of previous studies^1,4,10–17,20^. Prior to the between-group comparison, we checked whether tilt-dependent error (TDE), an index of SVBA shifts induced by body tilt (see Methods for details), depended on the direction of the body tilt or the lesion side. The TDEs were 29.0 ± 19.2° and 24.2 ± 17.4° for the leftward and rightward tilts in the control group, and 14.4 ± 17.3° and 17.5 ± 17.6° for the ipsilesional and contralesional tilts in the stroke group, respectively. Paired t-tests revealed that the TDEs did not differ significantly between the directions of body tilt both in the control (*t*_19_ = 1.84, *p* = 0.08, Cohen’s *d* = 0.41) and stroke groups (*t*_36_ = 1.33, *p* = 0.19, Cohen’s *d* = 0.18). Additionally, the TDEs did not differ significantly between patients with lesions in the left and right hemispheres for the stroke group (left-side lesion vs right-side lesion; 14.0 ± 16.1° vs. 14.6 ± 18.9° for the ipsilesional tilt and 16.2 ± 17.8° vs. 18.3 ± 17.2° for the contralesional tilt, respectively; both at *p* > 0.05). These results show that the TDEs were independent of both the direction of body tilt and the side of the lesion, allowing us to average the TDEs across tilt directions for each group and compare the mean TDEs between groups. A between-group comparison (t-test) revealed that the mean TDE was significantly larger in the control group (25.7 ± 17.1°) than the stroke group (15.9 ± 15.9°; *t*_55_ = 2.16, *p* = 0.03, Cohen’s *d* = 0.60; Fig. 1B). This result indicates that the accuracy of body axis orientation estimation was less affected by body tilt in patients with stroke than in healthy controls.

### Lesion analyses

**Figure 2:**
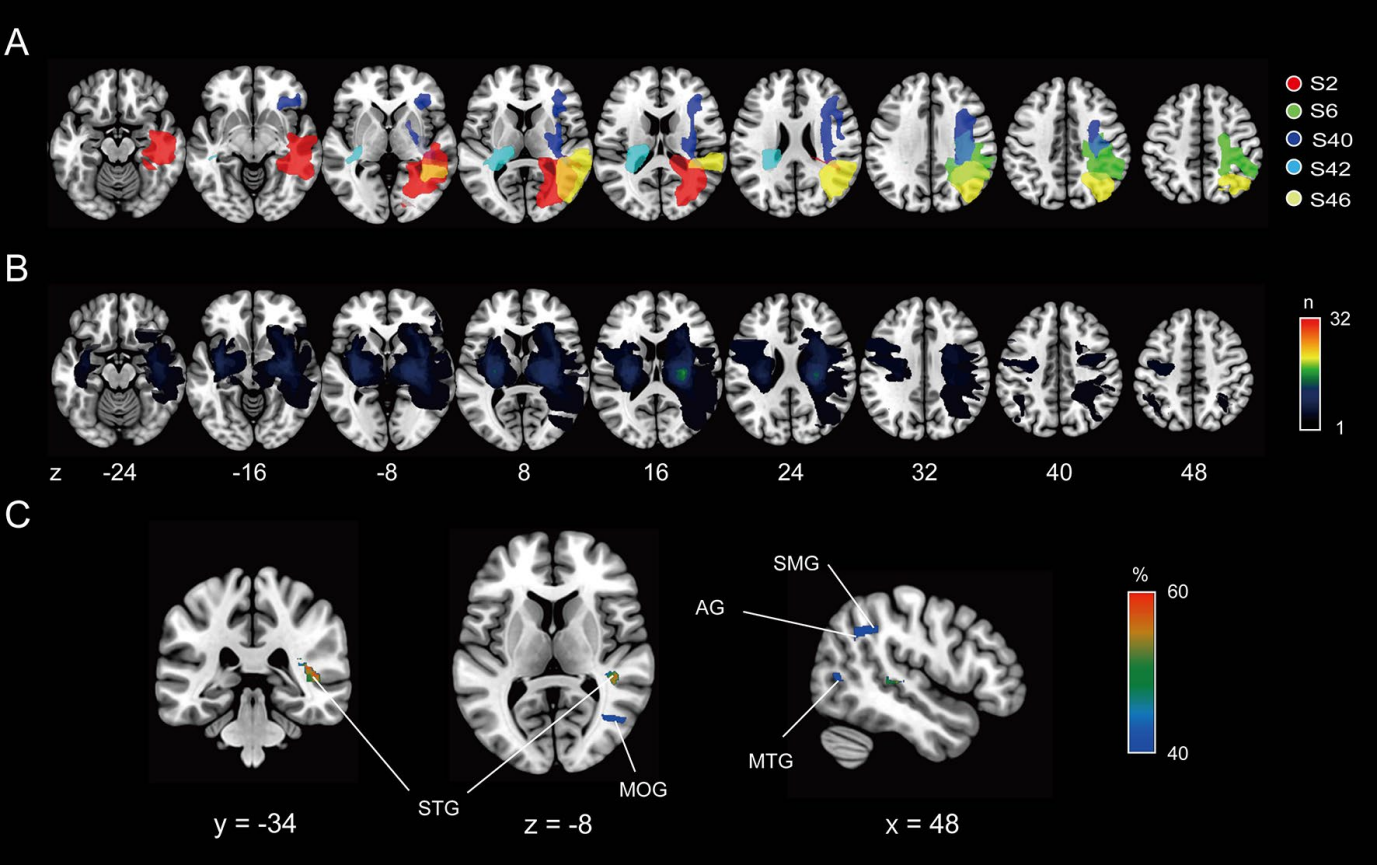
(**A**) Overlay lesion plot of patients in the abnormal TDE group (n = 5). The lesion extent for each patient is represented by different colours. (**B**) Overlapping lesion topographies of the patients in the normal TDE group (n = 32). Colour-coding represents the number of overlaying lesions per voxel. (**C**) Lesion subtraction map showing the cortical area more frequently damaged in the patients in the abnormal TDE groups compared to the patients in the normal TDE group. The colour bar indicates the percentage of overlap. For clarity, only voxels > 40% are shown.

To identify the lesion location responsible for the abnormally reduced TDE in stroke patients, we first conducted the lesion subtraction analysis. We classified the stroke patients into either the normal TDE group (*n* = 32, 18 right-side lesions, mean (± SD) TDE = 19.2 ± 14.8°) or abnormal TDE group [*n* = 5, ID = s2, s6, s40, s42, s46; 4 right-side lesions; mean (± SD) TDE = − 4.1 ± 1.8°] using the cut-off point [mean (25.7°) minus 1.5 × SD (17.1°) = 0.04°], defined by the TDE of healthy controls (see Methods for detail). Figure 2 illustrates each group’s overlay lesion images (each participant’s lesion location is shown for the abnormal TDE group; also see Fig. S1 showing a raw image of each participant) and the lesion subtraction plot. This map (only voxels with probability > 40% are displayed) showed that patients with an abnormally reduced TDE tended to have frequent damage centred on the right occipitotemporal cortex, specifically the superior (STG) and middle (MTG) temporal gyri, middle occipital gyrus (MOG), and inferior parietal lobules, including the supramarginal gyrus (SMG) and angular gyrus (AG). The peak voxel (probability = 67%) was located in the right MTG [Montreal Neurological Institute (MNI) x = 42, y = − 34, z = 8]. These results suggest that the lesions in the right occipitotemporal cortex can introduce the reduction in the tilt-dependent perceptual distortion of body axis orientation in stroke patients.

**Figure 3:**
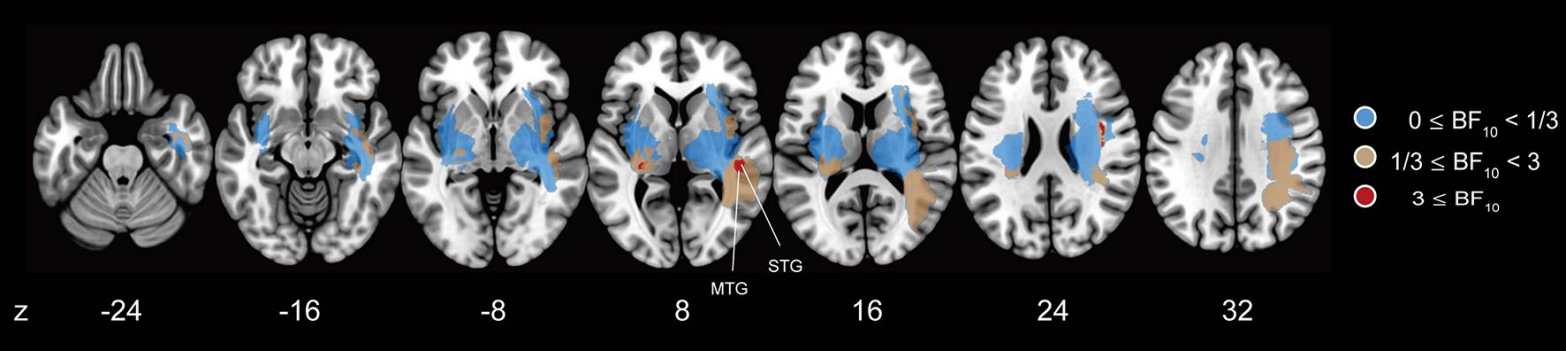
Bayes factor mapping results. Different colours are shown for each voxel with evidence level based on BF_10_. Uncoloured regions indicate areas where voxels were not included in the BLDI analysis.

To infer the statistical association between the lesion location and TDEs, we further performed a Bayesian lesion-deficit inference (BLDI) with Bayes factor mapping recently elaborated by Sperber and colleagues^21^. The authors showed that the BLDI would be well-suited, especially for the study with low power/sample (*n* < 50), compared with the frequentist lesion-mapping approach. Furthermore, the BLDI has the advantage of allowing us to discuss the strength of evidence for the null (H_0_: no association) or alternative hypotheses (H_1_: association between the presence of lesion and TDEs) based on the computed Bayes factor (BF_10_; Refs.^21,22^). In line with the subtraction analysis results, the voxel with substantial evidence for H_1_ (BF_10_ ≥ 3, red-coloured area) was found in the right MTG and STG with the right rolandic operculum and insula and left thalamus (Fig. 3 and also see Fig. S2 for detail). Voxels in the right MOG and inferior parietal lobes (SMG, AG) found to be associated with TDE abnormalities in lesion subtraction analysis were included in the area with 1/3 ≤ BF_10_ < 3 (ochre-coloured area) indicating no (or weak) evidence for H_0_ and H_1_.

### Relationship between tilt-dependent SVBA bias and dynamic postural ability

The relationship between tilt-dependent distortion of the body axis (TDE) and dynamic postural ability [dynamic Postural Assessment Scale for Stroke (PASS) score or lateral shift amplitude of centre of pressure (COP) in sitting] was evaluated in the stroke group. Pearson correlation analyses showed no significant correlation coefficient of TDE neither for the dynamic PASS score (*r* = -0.11, *p* = 0.51) or the COP lateral-shift amplitude (ipsilesional tilt, *r* = 0.24, *p* = 0.21; contralesional tilt, *r* = 0.21, *p* = 0.27). To assess the strength of evidence for the null hypothesis (H_0_) or alternative hypothesis (H_1_), we additionally conducted a Bayes factor (BF_10_) analysis with default Cauchy priors with a scale of 0.707 for Pearson correlation. BF_10_ was 0.53 between TDE and dynamic PASS, and 0.49 and 0.41 between the TDEs and the COP shift amplitude for ipsilesional and contralesional tilt, showing weak or no evidence for the H_0_^23^. We also checked the relationship between TDEs and the severity of unilateral spatial neglect (subjective midline deviation in the line bisection task). We found no significant correlation (*r* = − 0.17, *p* = 0.38) with weak or no evidence of the H_0_ (BF_10_ = 0.34).

We additionally performed partial correlation analyses with covariates of sensorimotor (Stroke Impairment Assessment Set: SIAS) and visuospatial function (line bisection task). Following the single correlation analysis, the partial correlation coefficient of TDE was insignificant for either the dynamic PASS score (partial *r* = 0.12, *p* = 0.50) or the COP lateral-shift amplitude (ipsilesional tilt, partial *r* = 0.16, *p* = 0.44; contralesional tilt, partial *r* = 0.24, *p* = 0.25; also see Table S1 for the partial *r* of each variable).

## Discussion

In the present study, using a neuropsychological approach, we aimed to determine the neural basis underlying the perceptual distortion of body axis orientation induced by body tilt. We investigated the relationship between the performance (accuracy) of the SVBA task and brain lesions in 37 patients with hemispheric stroke. The lesion subtraction analysis revealed an association between abnormalities in the tilt-dependent SVBA errors (TDE) and lesions in the right occipitotemporal cortex and inferior parietal lobule. Our results provide neuroanatomical evidence for body-centred spatial coding during the whole-body tilt.

### Reduced tilt-dependent SVBA bias in stroke patients

Behavioural analyses showed that the TDEs in the SVBA task were smaller in stroke patients than in healthy controls (Fig. 1B), as observed in a previous study^9^ in which although the authors did not directly assess the tilt-dependent SVBA bias). This indicates that the estimation of body axis orientation was “less” affected (i.e., accurate) by the change in body tilt angle in stroke patients. This trend is consistent with our VBM findings showing that the smaller the gray matter volume in specific brain regions, the smaller the TDE^16^, but appears counterintuitive according to the general assumption that brain damage should impair task performance (in this case, increasing TDE). One possible explanation is the contribution of the allocentric (gravity-based) representation of space to body-centred spatial processing. Recently, we investigated the association between the performance on the SVBA and subjective body tilt tasks, where the participants reported the body tilt angles relative to “gravity,” during whole-body tilt in the healthy individuals^17^. We found a strong positive correlation between the SVBA errors and reported body angles (i.e., the larger the estimated body tilt angle, the larger the SVBA bias in that direction) after adjusting for the actual body angles. This finding suggests that the brain at least partially relies on allocentric information involving body tilt orientation in space, even when estimating the egocentric (body-centred) orientation of the line. This idea is supported by psychophysical findings suggesting that egocentric and allocentric estimates are not entirely distinctly processed; instead, they share at least a partially common mechanism^24–26^. It is likely that the tilt-dependent SVBA bias reflects this characteristic of the brain and that brain lesions could impair the interaction between egocentric and allocentric spatial representations, resulting in reduced TDEs.

### Neural substrates underlying the tilt-dependent distortion of body-centred spatial coding

To identify the brain regions specifically involved in reduced TDE in patients with stroke, we performed a lesion subtraction analysis. We found that patients with an abnormally reduced TDE frequently had lesion extensions centred on the right occipitotemporal cortex, such as the right MOG, MTG, and STG (Fig. 2C). This finding supports our previous VBM study^16^ that showed a correlation between individual TDE and grey matter volume in the occipitotemporal regions, although the peak voxel was in the right MOG. Furthermore, previous neurophysiological studies have shown the activation of occipitotemporal regions, especially in the right hemisphere, when estimating a target location not only on egocentric coordinates, but also on allocentric coordinates^27–31^. Another study demonstrated that the modulation of egocentric spatial judgment by task-irrelevant allocentric visual cues correlated with cortical activity in the right MTG^32^. Together, these findings suggest that the right occipitotemporal region plays a key role in determining the interaction between egocentric and allocentric (gravity-based) reference frames.

We also found that reduced TDE was more frequently associated with lesions involving the right inferior parietal lobule, including the SMG and AG. The inferior parietal lobule is activated with right hemispheric dominance during both egocentric and allocentric tasks^27–31,33^. Moreover, a virtual lesion in the angular and/or supramarginal gyri induced by repetitive transcranial magnetic stimulation not only distorts the egocentric^34^ or allocentric (gravity-based) reference frames^35,36^, but also attenuates the influence of allocentric visual cues on egocentric movements^37^. Given these findings, the inferior parietal lobule, especially in the right hemisphere, is presumed to be involved in the functional link between the egocentric and allocentric reference frames and in controlling the contribution of gravity-based information to egocentric spatial coding, together with the right occipitotemporal cortex. Indeed, the human posterior lateral occipital area (hOc4lp), an extrastriate area nearly anatomically equivalent to the MOG, exhibits strong functional connectivity with the inferior parietal lobule during various types of visual tasks, including spatial discrimination^38^. The cortical regions identified in the present study may function as networks in body-centred spatial coding during the whole-body tilt.

However, the descriptive and statistical analyses (BLDI) results were inconsistent, suggesting that the relationship between the lesion and tilt-dependent SVBA bias needs to be carefully discussed. BLDI revealed the voxels with substantial evidence of the H_1_ (BF_10_ > 3) in the right STG and MTG in line with the descriptive finding; however, the voxels in the right MOG and inferior parietal lobules (SMG and AG) were found to be without evidence of the H_0_ and H_1_ (1/3 < BF_10_ < 3). This lack of evidence in the BLDI indicates the study’s low statistical power^21^. The TDE in the stroke group was smaller than in the control group; however, patients exhibiting abnormally reduced TDEs were rare (13% of the total), probably because the tilt-dependent SVBA bias inherently varies between individuals even in healthy controls^16^. This makes it difficult to statistically detect voxels associated with task performance^39^. In addition, the BLDI showed an association (BF_10_ > 3) with TDEs in cortical regions such as the left thalamus and right rolandic operculum and insula in contrast to the descriptive analysis. These results may be supported by previous studies reporting the involvement of the lesions in these areas in inaccurate spatial orientation, such as visual verticality in the body upright^40,41^ or tilted positions^42^. However, drawing conclusions remains challenging because only one patient with damage in these areas was included in the abnormal TDE group (Fig. 2A), and BLDI is more prone to false positives than frequentist mapping analysis^21^. Further studies with larger sample sizes are needed to address these issues.

We should also note two limitations of our study. First, the distribution of right hemisphere lesions versus left hemisphere lesions was asymmetrical (Fig. 2). This asymmetry can be attributed, at least in part, to the difficulties in task comprehension for patients with aphasia caused by left hemisphere damage, resulting in the exclusion of patients with left hemisphere damage from our study. To investigate the involvement of the bilateral occipitotemporal cortex in TDE, as demonstrated by our VBM study^16^, it is necessary to conduct a more detailed examination explicitly focusing on patients with left hemisphere damage. Second, the perception of visual vertical was not evaluated in stroke patients. Similar to the assessment of body axis perception (SVBA), the subjective visual vertical (SVV), which can be quantified by asking participants to align a visual segment parallel to "gravity," is known to be influenced by body tilt^43,44^. Moreover, several studies have reported a significant correlation between SVV and SVBA performance when the body is laterally tilted^9,12,45^. These facts suggest that the identified brain regions associated with SVBA performance (TDE) may also be related to visual vertical perception. Indeed, previous studies have shown that lesions in regions common with (but slightly more lateral than those in) the present study, such as the MTG, STG, and inferior parietal lobule, are associated with inaccuracy in the SVV task^46,47^. Future neuropsychological studies involving both SVV and SVBA assessments will contribute to a better understanding of the shared and/or distinct neural substrates underlying these spatial orientation tasks.

### Absence of association between the tilt-dependent distortion of body axis perception and dynamic postural ability

The secondary purpose of this study was to investigate the relationship between the perception of body axis orientation during whole-body tilt and dynamic postural ability in patients with stroke. In contrast to previous studies showing a correlation between the perceived body axis and static postural orientation^1,20^, we found no significant association between the TDEs in the SVBA task and the dynamic PASS score or COP lateral-shift amplitude. These results suggest a lower contribution of the perceived body axis as a reference frame to dynamic postural control; however, this needs to be further validated with a larger sample considering Bayesian statistics results showing no/weak evidence for the H_0_. Previous clinical studies have shown that the performance on a task involving the estimation of gravitational direction, such as the visual or postural vertical, is related to dynamic balance abilities in stroke patients^48–50^. The difference between the previous and current findings allows us to consider that balance control in the gravitational field might rely largely on the gravity-based reference frame rather than on the egocentric reference frame. Because the gravitational torque acting on the body changes depending on the body orientation relative to gravity, the accurate perception of body orientation in the gravity-based reference frame is likely crucial for postural control against gravity^51,52^. Rather, the egocentric (body-centred) reference frame would be more relevant for goal-directed movements (e.g., reaching) as it requires the localization and encoding of target locations relative to one's own body for planning^15,53^. Numerous experimental findings suggest that the brain is likely to flexibly determine which reference frames to use based on task demands or the states of the body and environment^54,55^. This mechanism may underlie the differential contribution of the perceived body axis to the static and dynamic postural abilities in patients with stroke.

## Conclusion

The present results show that brain lesions in the right occipitotemporal cortex, such as superior and middle temporal gyri, are associated with the abnormality of the body tilt-induced bias of perceived body axis orientation in hemispheric stroke patients. This finding highlights a vital role of these areas in body-centred spatial coding, particularly when the head and/or body is tilted relative to gravity. Future research using neuromodulation techniques is needed to provide more direct evidence for the involvement of the identified regions in the perception of body axis orientation.

## Methods

### Participants

This study was approved by the Ethical Committee of Hamamatsu City Rehabilitation Hospital and Hamamatsu University School of Medicine and was conducted in accordance with the Declaration of Helsinki (2013). All participants provided written informed consent before participation.

Forty patients with their first stroke (cerebral haemorrhage or cerebral infarction) admitted to Hamamatsu City Rehabilitation Hospital between April 2019 and February 2021 were recruited. The inclusion criteria were as follows: (1) age between 20 and 89 years, (2) right-handedness before the onset of stroke, (3) no bilateral lesions, cerebellum or brainstem, (4) no organic vestibular disease, dizziness or vertigo, strong motion sickness, and visual deficit (hemianopia), (5) no severe musculoskeletal disorder, (6) no history of organic disease in the nervous system (spinal injury), (7) behavioural tests or head CT/MRI scans within 3 months after onset, (8) preserved capacity to sit without support for over 30 s. In addition, patients with difficulties in understanding the task requirements (such as cognitive deficits or aphasia) were excluded. The mean (± SD) time interval of the spatial orientation assessment (SVBA task) from the onset of stroke was 51.0 ± 21.3 days (ranged 15—107 days). Three participants were excluded because severe cerebral atrophy and bilateral lesions were detected. Accordingly, the data for 37 patients (mean ± SD, 62.1 ± 12.1 years; 16 women; 22 right-side strokes) was finally applied to the analyses for spatial orientation performance. In the stroke group, data could not be acquired for nine patients: seven in the postural assessments and two in the evaluation for unilateral spatial neglect. Hence, data from 30 and 28 patients were applied to evaluate the relationship between SVBA task performance and dynamic postural ability in the Pearson and partial correlation analyses, respectively. Twenty healthy right-handed controls (65.3 ± 9.8 years; 11 women), age-matched with the stroke group, were recruited only for the assessments of spatial orientation (see Supplementary Data for individual data).

The sample size for the stroke group was calculated using G* Power software (version 3.1.9.2; Heinrich-Heine-Universität, Düsseldorf, Germany). Effect size was set at Cohen’s *f*^2^ = 0.51 for inter-subject correlation analyses(*α* = 0.05, 1 − *β* = 0.8, number of predictors = 5) for a multiple regression analysis with reference to a previous study^1^ on human spatial orientation. The dropout rate was set at 0.2, assuming that some participants would be unable to complete the series of assessments. The study protocol was pre-registered in the University Hospital Medical Information Network (registration number: UMIN000036407).

### CT scans and MRI

To analyse the location and extension of the lesion in each patient, head computed tomography (CT) scans (34 patients) or magnetic resonance imaging (MRI; three patients) were performed. Mean (± SD) time interval between CT or MRI scans and the onset of stroke was 28.2 ± 15.6 days (ranged 8 to 72 days). CT scans were acquired using a BrightSpeed Elite SD (GE Healthcare, Japan) with a slice thickness of 5 mm and an interslice gap of 5 mm (number of slices = 20). T1-weighted sequences were used for the MRI scanning. The T1-weighted images were acquired using Optima MR360 Advance 1.5 T (GE Healthcare, Japan) with the following parameters: repetition time = 550 ms, echo time = 11 ms, flip angle = 70°, percent phase of field of view = 80, slice thickness = 5 mm, interslice gap = 2 mm, number of slices = 28). The boundaries of the lesions were manually delineated on CT or MRI scans on a slice-by-slice basis by one experimenter (A. N.) who was blinded to the behavioural performances and attributes of the patients, using the MRIcron software package (v.1.0.20190902). Lesion maps were converted into a 3-dimensional volume of interest. Subsequently, lesion volume and anatomical scans were normalized to the MNI space with 1 mm × 1 mm × 1 mm resolution based on the brain templates of healthy individuals via the Clinical Toolbox^56^ in MATLAB version 9.8.0 (R2020a).

### Subjective visual body axis (SVBA) task

**Figure 4:**
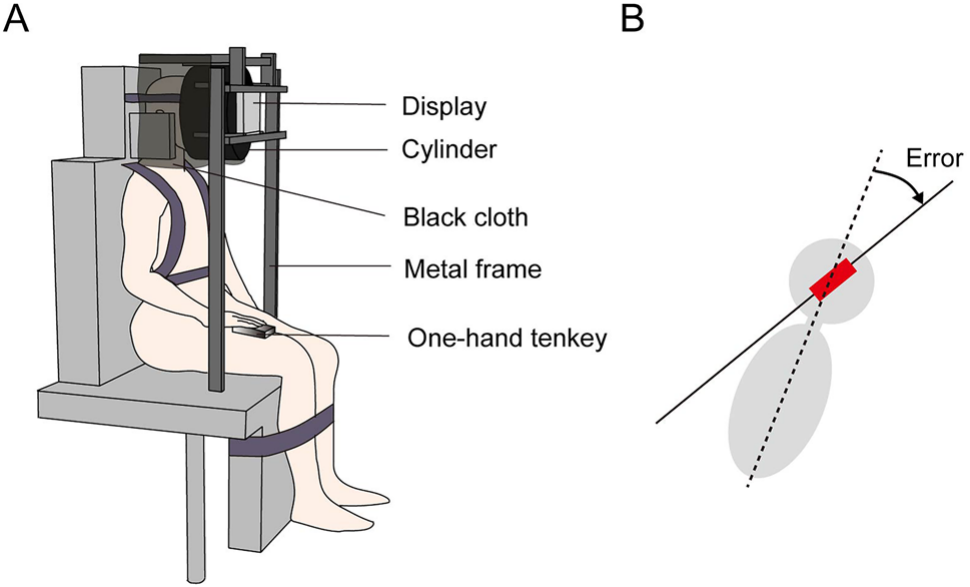
(**A**) Experimental setup. (**B**) Schematic illustration of estimation error calculation in the SVBA task. Participants aligned the visual line (denoted as a red line) along the perceived direction of the body longitudinal axis (SVBA). SVBA errors were defined as the angular deviation of the visual line from the actual body axis (denoted as a dashed line).

The perception of body axis orientation was assessed using the SVBA task, as in our previous studies^4,16^. Participants sat on a tilting chair (SP-PS100-Z, Pair Support, Japan) that could be rotated on the frontal plane at a maximum velocity of 0.69°/s, and their head, trunk, and legs were secured to the seat with bands and a seatbelt. A 10.1-inch display (width: 25.5 cm, height: 16.4 cm; AUS50024E, ELECROW, China) was placed in front of the head. A black cylinder (26 cm in diameter) with a hole (11.0 cm in diameter) on one side was inserted between the head and display, and the upper sides of the cylinder and head were covered with a black cloth (Fig. 4A). This method prevented participants from using visual cues (e.g., the edge of the display or pillars of the room) other than the visual line for the task. In the SVBA task, the participants were instructed to adjust a visual segment (4.6 cm in length) on the display along the perceived head/body longitudinal axis. The visual line can be rotated in 0.5° increments by manipulating a one-hand tenkey (nump-1421, Rottay, United States) with a non-paralyzed hand for patients or a digital controller (F310r; Logitech, Lausanne, Switzerland) for controls. The initial orientation of a visual line was pseudo-randomly set at ± 45°, ± 60°, or 90° with respect to the participants’ longitudinal axis. Participants consecutively performed the SVBA task for 10 trials each in an upright position (0°) or rightward or leftward tilted position at 10° (i.e., 30 trials in total). Time limits were not set for SVBA tasks. The initial body angle was 0°, and the other angles were presented in a randomized order for each participant.

### Assessment of postural ability and other neurological functions

Postural ability was assessed using the Postural Assessment Scale for Stroke (PASS), comprising 12 items with scores ranging from 0 to 36 points (good performance). PASS comprises static (posture maintenance) and dynamic (posture transformation) factors. In this study, we focused on the relationship between the perception of body axis orientation during whole-body tilt and dynamic postural ability. Therefore, we used only the dynamic PASS score for the partial correlation analysis (see Data analysis for details).

Additionally, the COP in the sitting position was measured using a rectangular force platform (43.0 cm × 37.0 cm) attached to a Digital Mirror system (Panasonic, Japan) at a sampling rate of 60 Hz. Each participant was seated on a chair (height, 40 cm) with a force plate placed between the buttocks and the chair surface. The sitting position was individually set so that the soles of the feet were on the floor and the centre of the buttocks was aligned with that of the force plate. First, we assessed the COP position during static sitting by asking the patients to maintain an upright position for 30 s with their eyes closed and both upper limbs crossed in front of their chests. Next, the patients were asked to tilt their trunk toward the ipsilesional or contralesional sides as much as possible with their eyes closed. Each tilt direction was performed twice and the most deviated COP position in the mediolateral direction was evaluated. The amount of COP lateral shift on each side was calculated by subtracting the COP position in the static sitting position from the maximum COP position for the trunk lateral tilt.

The motor and sensory functions of each patient were quantified as the sum of seven motor-related items (including two items for trunk function) and four sensory-related items of the Stroke Impairment Assessment Set (SIAS). The degree of visuospatial function (spatial neglect) was assessed by the line bisection task in which participants were asked to determine the midpoints of 17 horizontal lines with various lengths (range 7.3–14.9 cm). The mean deviation of the subjective midpoints from the true midpoints was used for the partial correlation analyses. In addition, the degree of pushing behaviour toward the contralesional side was evaluated using the Scale for Contraversive Pushing (SCP), which ranged from 0 to 6. The data for each assessment in the stroke group are shown in Table 1 (group-mean) and Table S2 (by lesion side).

**Table 1:** Group data for neurological and postural assessments in stroke group.

SIAS motor (0–31, au)	19.9 ± 7.7 (0–31)
SIAS sensory (0–12, au)	7.7 ± 4.1 (0–12)
Line bisection tests (mm)	2.2 ± 6.5 (− 0.4–3.3)
SCP (0–6, au)	0.3 ± 0.8 (0–3.0)
PASS dynamic (0–21, au)	19.0 ± 3.3 (10–21)
COP upright position (cm)	4.7 ± 10.2 (− 13.0–27.8)
COP lateral-shift amplitude	
Ipsilesional side (cm)	70.8 ± 22.8 (37.8–121.0)
Contralesional side (cm)	73.4 ± 21.8 (25.5–103.5)

Mean ± SD (range). “COP upright position” indicates the mean position of centre of pressure (COP) in the mediolateral plane during upright sitting. “COP lateral-shift amplitude” indicates maximum amount of COP shifts during the trunk tilt toward ipsilateral or contralateral sides. The positive signs of the data for the line bisection test and COP assessment in the upright position corresponded to shifts toward the ipsilesional side. The positive sign of the COP lateral shift amplitude corresponds to the COP shift toward the side of the trunk tilt.

*au* arbitrary units, *SIAS* stroke impairment assessment set, *SCP* scale for contraversive pushing, *PASS* postural assessment scale for stroke, *SD* standard deviation.

### Data analysis

The SVBA angle, the angular difference between the subjective and actual directions of the body’s longitudinal axis, was evaluated in each trial (Fig. 4B). The SVBA angles were averaged across ten trials for each body angle. For the control participants, the positive and negative values of the SVBA angles correspond to a rightward and leftward bias, respectively. For stroke patients, the positive–negative sign of the SVBA angles was transformed according to the lesion side so that the positive and negative values correspond to an ipsilesional and contralesional bias, respectively. To quantify the perceptual distortion of body axis orientation induced by whole-body tilt, TDEs for the left and right (ipsilesional and contralesional sides for stroke patients) body tilts were computed as follows:

$${TDE}_{leftward \,(ipsilesional)} ={SVBA }_{leftward\, \left(ipsilesional\right)}- {SVBA}_{upright}$$

$${TDE}_{rightward\, (contralesional)} ={SVBA }_{rightward\, \left(contralesional\right)}- {SVBA}_{upright}$$

The positive–negative sign of TDE for the leftward tilt (ipsilesional side for stroke patients) was reversed, with positive values corresponding to SVBA shifts in the direction of body tilt. As a result, a large TDE indicated a large SVBA shift in the direction of body tilt.

To evaluate the overall impact of hemispheric stroke on tilt-dependent perceptual distortion of body axis orientation, TDEs were compared between the stroke and control groups. There was no significant difference in TDEs between the tilt directions (i.e., leftward tilt vs. rightward tilt for controls and contralesional tilt vs. ipsilesional tilt for patients) in both groups (see “Results”). Thus, the mean values of the TDEs in the two tilt directions were used as the representative values for each participant. We then compared the mean TDEs between the groups. The mean TDEs were normally distributed across participants in both the control and stroke groups (Shapiro–Wilk test, both at *p* > 0.05). A t-test (two-tailed) was used to compare TDE between groups.

To identify the brain region related to the abnormally reduced TDE in stroke patients, we performed lesion subtraction analysis^57^. To classify the patients into two groups, with or without the *abnormally* reduced TDE, we defined the cut-off as 1.5 SD below the mean TDE of the control group. This cut-off point is relatively conservative and is often used in neuropsychological studies involving perceptual and cognitive performance (e.g., Refs.^58,59^). As a result, five patients were assigned to the “abnormal” TDE group, while 32 patients to the *normal* TDE group. For each group, lesion images were superimposed using the MRIcron software, and the overlapping lesion images of the normal TDE group were subtracted from those of the abnormal TDE group. The created lesion subtraction map highlights the lesion location that is frequently observed in patients with the abnormally reduced TDE.

For statistical inference regarding the association between lesion location and TDEs, we further performed a Bayesian lesion-deficit inference (BLDI) using a Bayesian Lesion-Deficit Inference Toolkit (Ref.^21^; https://github.com/ChrisSperber/BLDI) implemented in R (R Core Team, 2021). A Bayesian two-sample t-test comparing the TDEs of patients with lesions to that of patients without lesions was performed in each voxel, and then the resulting voxel-wise Bayes factor (BF_10_) showing the strength of evidence for null (H_0_) or alternative hypotheses (H_1_) were mapped to the MNI brain space. The analysis did not include total lesion size as a covariate given a recent study^60^ showing that controlling for lesion size would be problematic, especially for a study with low statistical power due to a small sample size or noisy behavioural assessment. Only voxels damaged in at least three patients were analysed, including 110,689 voxels in the BLDI analysis. BF_10_ ≥ 3 indicates evidence greater than or equal to substantial for H_1,_ 1/3 ≤ BF_10_ < 3 indicates no evidence for H_0_ and H_1_, and BF_10_ < 1/3 indicates evidence greater than or equal to substantial for H_0_^23^.

The association between the tilt-dependent perceptual distortion of body axis orientation (TDE) and dynamic postural ability (dynamic PASS score or COP shift) was assessed using Pearson and partial correlation analyses. To assess the correlation with the dynamic PASS score, the average TDE values for the ipsilesional and contralesional tilts were used. Correlations between lateral COP shifts and TDEs were evaluated for each tilt side (contralesional or ipsilesional). In partial correlation sanalysis, we set the SIAS-motor and SIAS-sensory points and the deviation in the line bisection test as covariates. The significance level was set at *p* < 0.05. All statistical analyses of behavioural data were performed using JASP software (version 0.16.4.0).

## Supplementary Information

Supplementary Information 1.

Supplementary Information 2.

## Data Availability

All data generated or analysed during this study are included in this published article and its supplementary information files.

## Abbreviations

AG: Angular gyrus
BF: Bayes factor
BLDI: Bayesian lesion-deficit inference
COP: Centre of pressure
CT: Computed tomography
hOc4lp: Human posterior lateral occipital area
MOG: Middle occipital gyrus
MNI: Montreal Neurological Institute
MRI: Magnetic resonance imaging
MTG: Middle temporal gyrus
PASS: Postural assessment scale for stroke
SCP: Scale for contraversive pushing
SD: Standard deviation
SIAS: Stroke impairment assessment set
SMG: Supramarginal gyrus
STG: Superior temporal gyrus
SVBA: Subjective visual body axis
SVV: Subjective visual vertical
TDE: Tilt-dependent error
VBM: Voxel-based morphometry
