# Supplementary material for: Neuroanatomy of reduced distortion of body-centred spatial coding during body tilt in stroke patients
Source: Sci Rep. 2023 Jul 22;13:11853. doi: 10.1038/s41598-023-38751-0 (PMC10363170; doi:10.1038/s41598-023-38751-0)

Supplementary material

Neuroanatomy of reduced distortion of body-centred spatial coding during body tilt in stroke patients

Keisuke Tani[1,2*], Shintaro Iio[3], Masato Kamiya[3], Kohei Yoshizawa[3], Takashi Shigematsu[4], Ichiro Fujishima[4], Satoshi Tanaka[1]

[1] Laboratory of Psychology, Hamamatsu University School of Medicine, Hamamatsu, Shizuoka 431-3192, Japan

[2] Faculty of Psychology, Otemon Gakuin University, Ibaraki, Osaka 567-8502, Japan

[3] Department of Rehabilitation, Hamamatsu City Rehabilitation Hospital, Hamamatsu, Shizuoka 433-8511, Japan

[4] Department of Rehabilitation Medicine, Hamamatsu City Rehabilitation Hospital, Hamamatsu, Shizuoka 433-8511, Japan

***Corresponding Author**

Keisuke Tani, Ph.D

Faculty of Psychology, Otemon Gakuin University, 2-1-15 Nishi-Ai, Ibaraki, Osaka 567-8502, Japan

Email: keisuketani.pt@gmail.com

Table S1: Partial r value of each variable

	Dynamic PASS	COP shift amplitude (ipsilesional tilt)	COP shift amplitude (contralesional tilt)
TDE	0.12	0.16	0.27
SIAS-motor	0.70***	0.36	0.14
SIAS-sensory	-0.30	-0.09	0.29
Line bisection	-0.02	-0.04	-0.02

PASS, Postural Assessment Scale for Stroke; SVBA, subjective visual body axis; SIAS, Stroke Impairment Assessment Set; TDE, tilt-dependent error. ***: $p < 0.001$

Table S2: Data from neurological and postural assessments of patients with left- and right-hemispheric lesions.

		Left-side lesion	Right-side lesion	*p* value
SIAS motor (0 — 31, au)		20.7 (9.1)	19.6 (7.3)	0.38
SIAS sensory (0 — 12, au)		4.3 (1.6)	4.0 (2.1)	0.36
Line bisection tests (mm)		0.1 (0.2)	0.4 (0.9)	0.19
SCP (0 — 6, au)		0.1 (0.28)	0.4 (0.83)	0.10
PASS dynamic (0 — 21, au)		19.5 (3.5)	18.5 (3.1)	0.39
COP upright position (cm)		2.8 (8.6)	6.3 (11.5)	0.36
COP lateral-shift amplitude	ipsilesional side (cm)	77.6 (24.7)	64.8 (19.9)	0.04
	contralesional side (cm)	81.9 (21.6)	66.0 (19.8)	0.13

Mean (SD). "COP upright position" indicates the mean position of centre of pressure (COP) in the mediolateral plane during upright sitting. "COP lateral-shift amplitude" indicates maximum amount of COP shifts during the trunk tilt toward ipsilateral or contralateral sides. The positive signs of the data for the line bisection test and COP assessment in the upright position corresponded to shifts toward the ipsilesional side. The positive sign of the COP lateral shift amplitude corresponds to the COP shift toward the side of the trunk tilt. T-tests (two-tails) were used for between-group comparison. au, arbitrary units; SIAS, Stroke Impairment Assessment Set; SCP, Scale for Contraversive pushing; PASS, Postural Assessment Scale for Stroke. SD, standard deviation.

Figure S1: Raw image data (CT) of each participant (ID = s2, s6, s40, s42, s46) in the abnormal TDE group. Z values represent the height of each slice on the scanner coordinates.

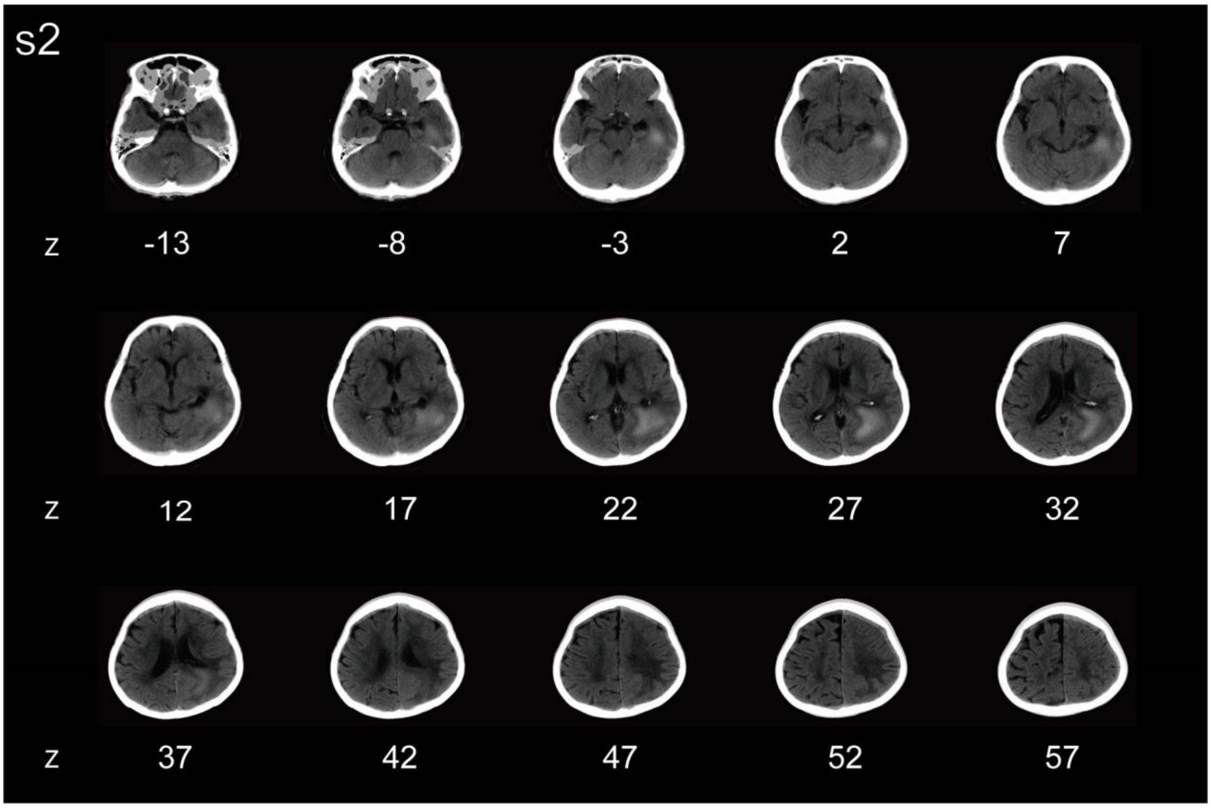

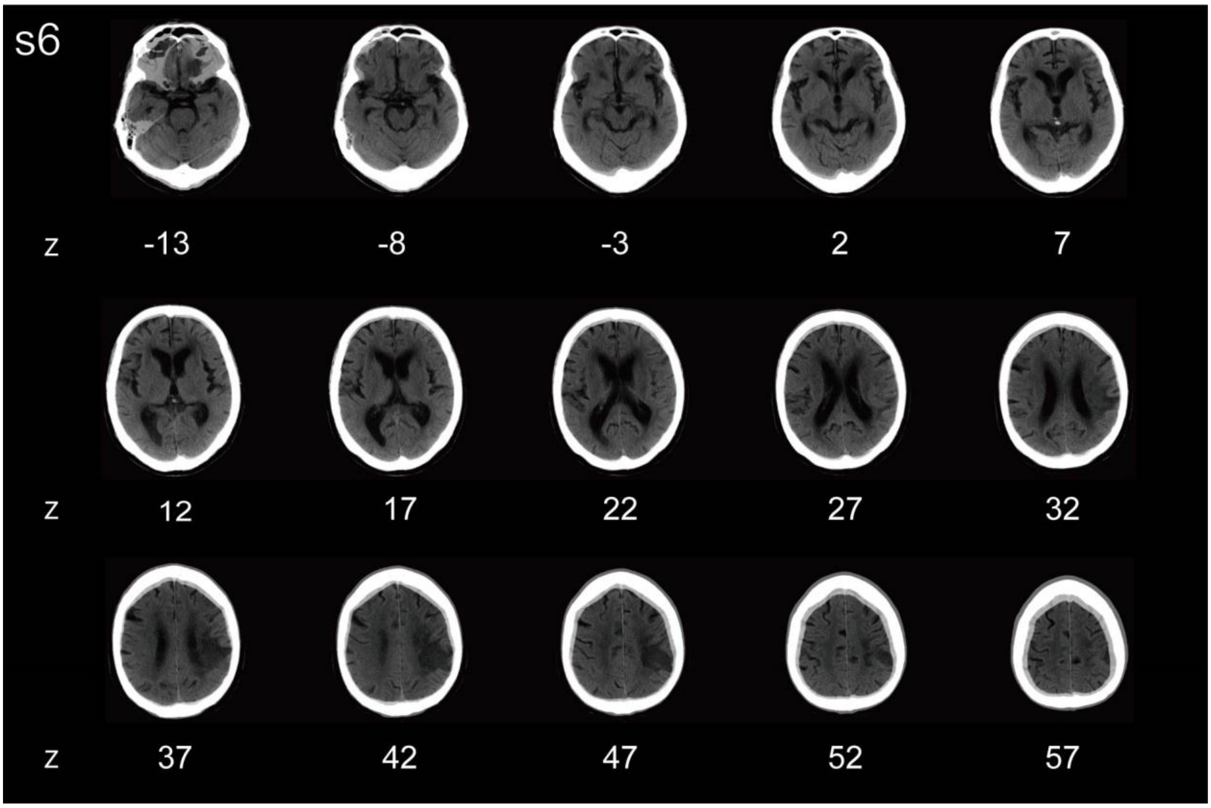

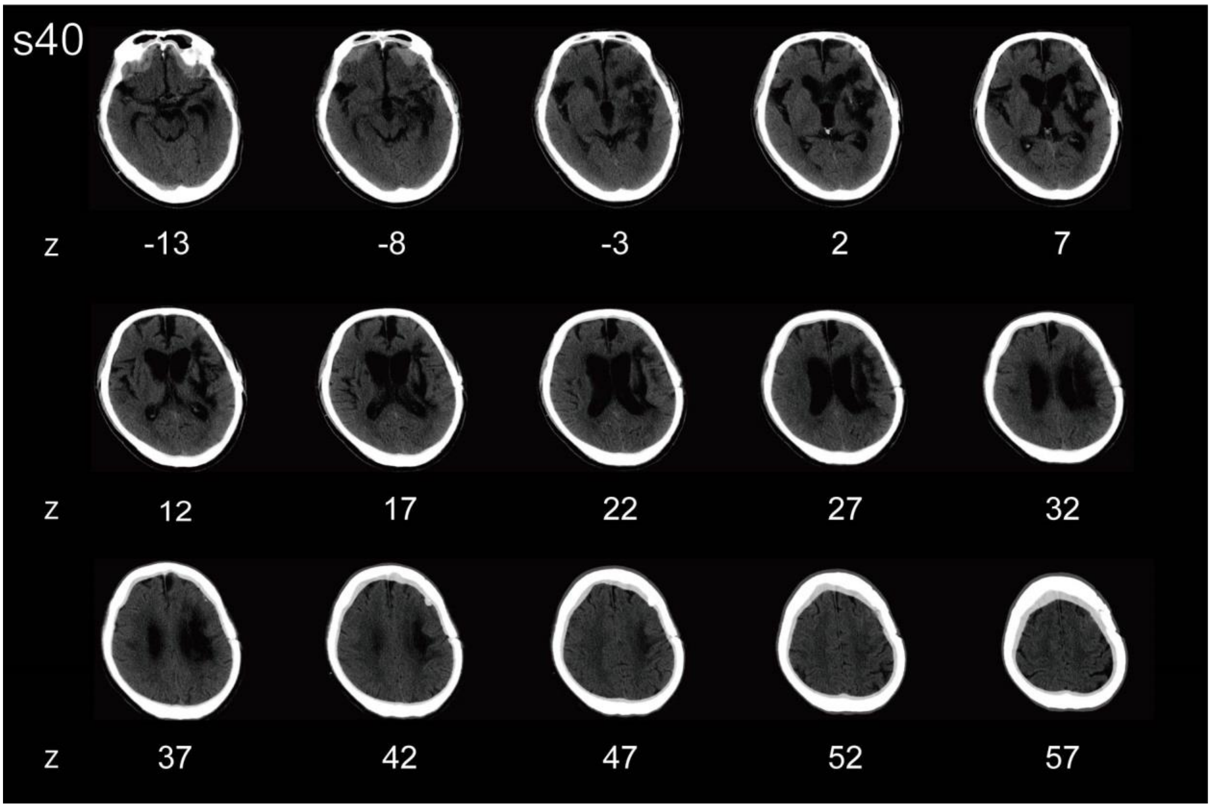
s40
z
-13
-8
-3
2
7
z
12
17
22
27
32
z
37
42
47
52
57

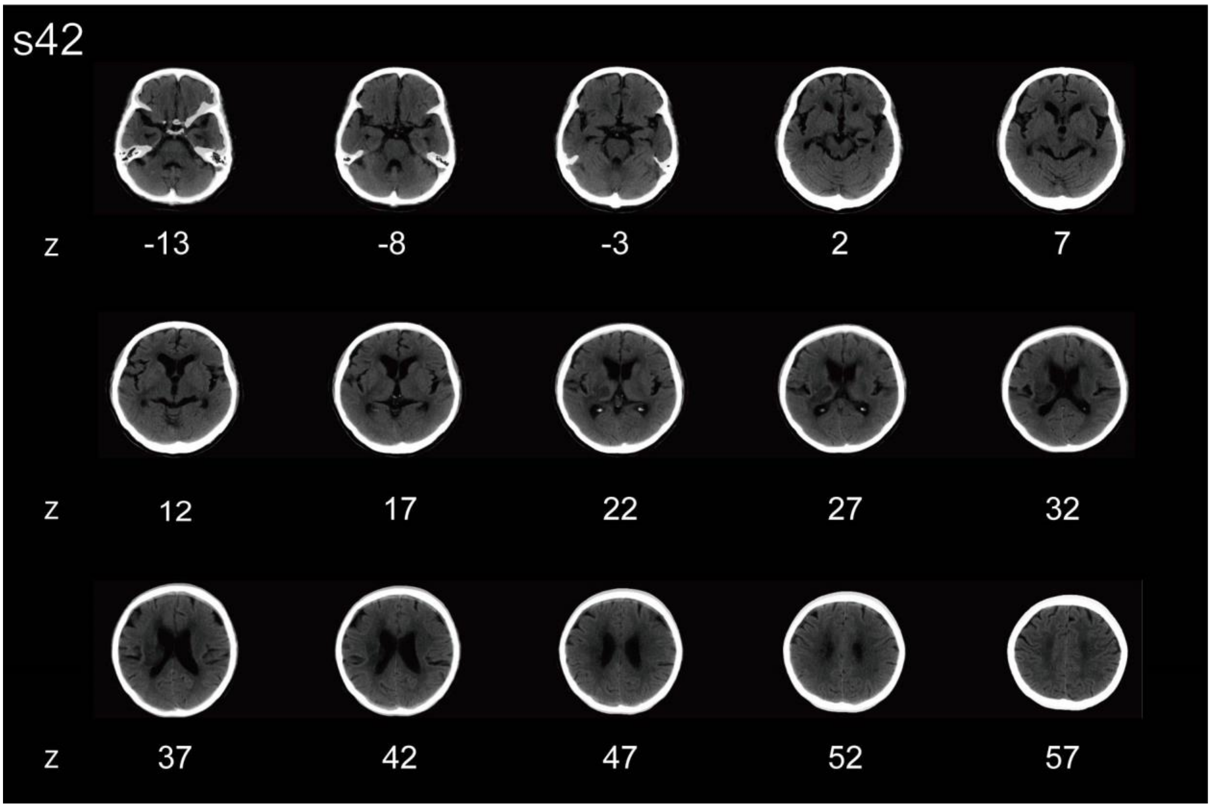
s42
z
-13
-8
-3
2
7
z
12
17
22
27
32
z
37
42
47
52
57

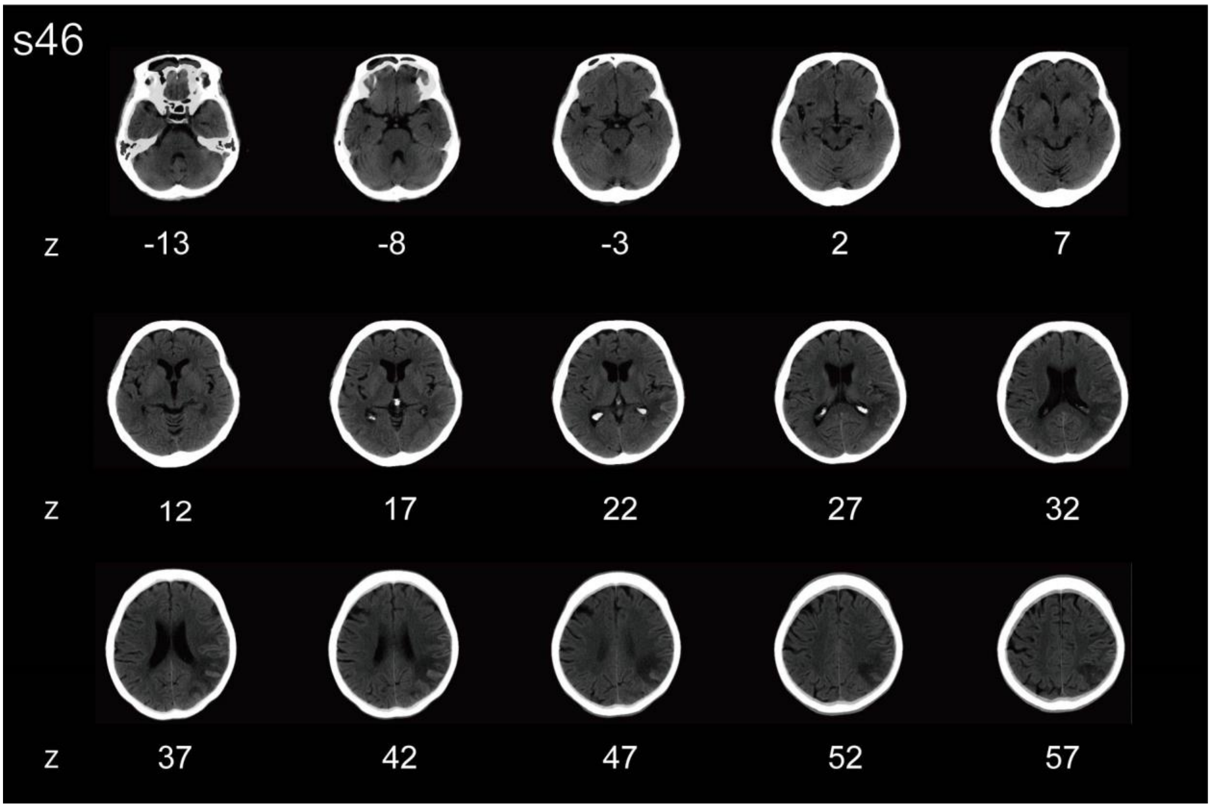

Figure S2: Detailed results of Bayes factor mapping (per 4 mm slice). Different colours are shown for each voxel with evidence level based on BF_{10}. Uncoloured regions indicate areas where voxels were not included in the BLDI analysis.

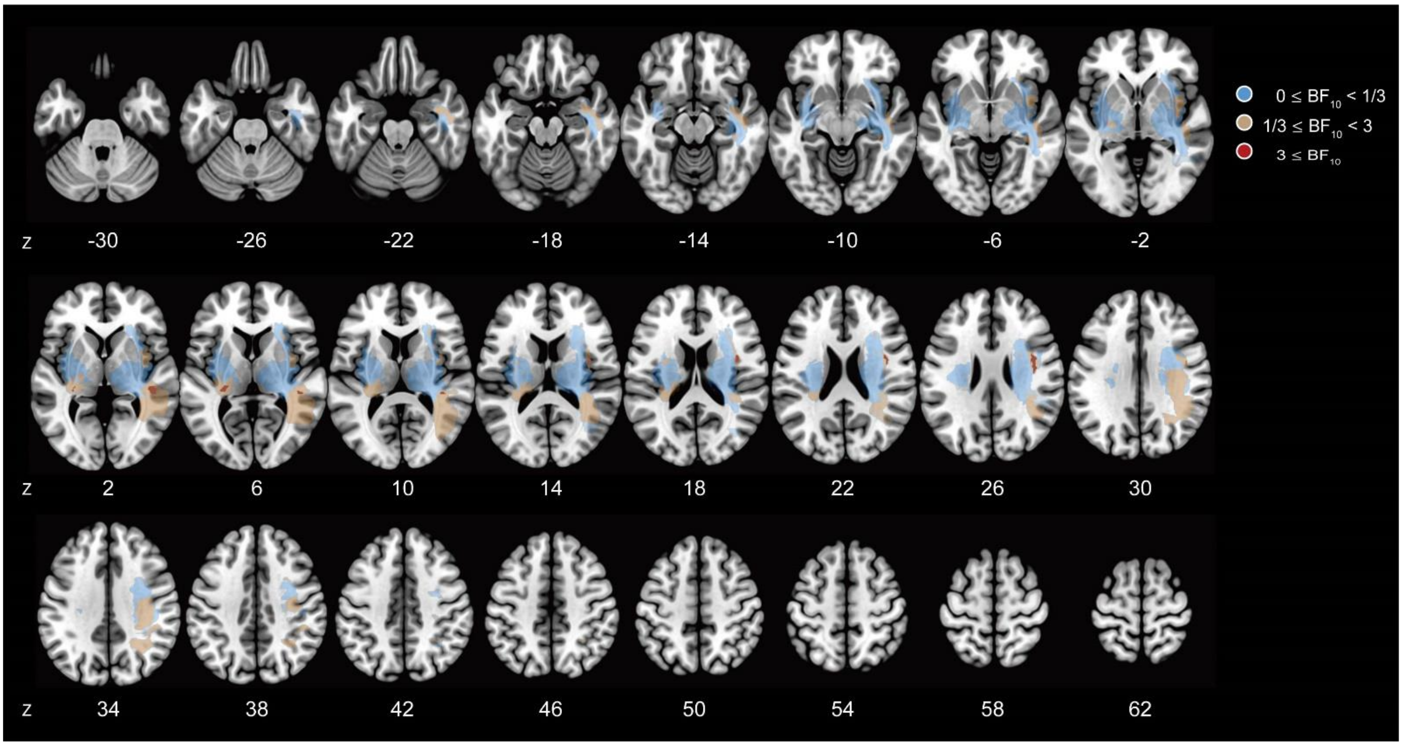

Supplement: Supplementary file 2 — Supplementary Information 2. [file 41598_2023_38751_MOESM2_ESM.pdf]
